# Structural Model and Spin-Glass Magnetism of the Ce_3_Au_13_Ge_4_ Quasicrystalline Approximant

**DOI:** 10.1021/acs.inorgchem.0c03430

**Published:** 2021-02-03

**Authors:** Pascal Boulet, Marie-Cécile de Weerd, Mitja Krnel, Stanislav Vrtnik, Zvonko Jagličić, Janez Dolinšek

**Affiliations:** † Institut Jean Lamour, UMR 7198, CNRS, Université de Lorraine, Campus Artem, 2 allée André Guinier, BP 50840, 54011 Nancy Cedex, France; ‡ J. Stefan Institute, Jamova 39, SI-1000 Ljubljana, Slovenia; § Institute of Mathematics, Physics and Mechanics, Jadranska 19, SI-1000 Ljubljana, Slovenia; ∥ Faculty of Civil and Geodetic Engineering, University of Ljubljana, Jamova 2, SI-1000 Ljubljana, Slovenia; ⊥ Faculty of Mathematics and Physics, University of Ljubljana, Jadranska 19, SI-1000 Ljubljana, Slovenia

## Abstract

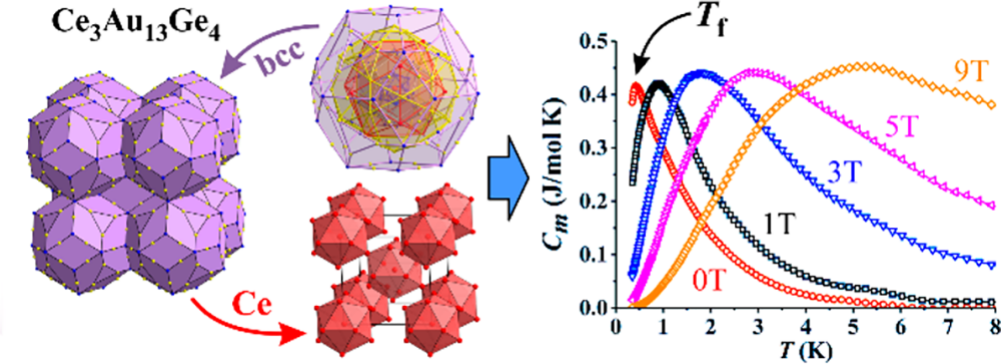

In a search for unconventional heavy-Fermion compounds with the localized 4f moments distributed quasiperiodically instead of a conventional distribution on a regular, translationally periodic lattice, we have successfully synthesized a stable Ce_3_Au_13_Ge_4_ Tsai-type 1/1 quasicrystalline approximant of the off-stoichiometric composition Ce_3+*x*_Au_13+*y*_Ge_4+*z*_ (*x* = 0.17, *y* = 0.49, *z* = 1.08) and determined its structural model. The structure is body-centered-cubic (bcc), with space group *Im*3̅, unit cell parameter *a* = 14.874(3) Å, and Pearson symbol *cI*174, and can be described as a bcc packing of partially interpenetrating multishell rhombic triacontahedral clusters. The cerium sublattice, corresponding to the magnetic sublattice, consists of a bcc packing of Ce icosahedra with an additional Ce atom in a partially occupied site (occupation 0.7) at the center of each icosahedron. The measurements of its magnetic properties and the specific heat have demonstrated that it is a regular intermetallic compound with no resemblance to heavy-Fermion systems. The partially occupied Ce2 site in the center of each Ce1 icosahedron, the mixed-occupied Au/Ge ligand sites between the Ce2 and Ce1 atoms, and the random compositional fluctuations due to nonstoichiometry of the investigated Ce_3+*x*_Au_13+*y*_Ge_4+*z*_ alloy introduce randomness into the Ce magnetic sublattice, which causes a distribution of the indirect-exchange antiferromagnetic interactions between the spins. Together with the geometric frustration of the triangularly distributed Ce moments, this leads to a spin-glass phase below the spin freezing temperature *T*_f_ ≈ 0.28 K.

## Abstract 2

A stable Ce_3_Au_13_Ge_4_ Tsai-type 1/1 quasicrystalline approximant was synthesized, and its structural model and magnetic properties were determined. Randomness and geometric frustration within the Ce sublattice lead to a spin-glass phase below 0.28 K.

## 1 Introduction

Despite the great success of the assumption to treat conduction electrons in metals as noncorrelated (i.e., noninteracting) in many areas of solid-state physics, there also exist materials with properties that cannot be described by the theory of a free-electron gas but are essentially determined by strong electronic correlations. The materials are denoted as strongly correlated electron systems, and the phenomena originating from the electronic correlations include superconductivity, colossal magnetoresistance, fractional quantum Hall effect, heavy-Fermion (HF) behavior, and quantum criticality. HF metals are characterized by a dramatic increase of the effective mass of charge carriers at low temperatures, which may reach up to 1000 times the mass of a free electron. This phenomenon is caused by the Kondo effect, where the free electrons are coupled to the localized magnetic moments originating from unpaired electrons that are fixed to the crystal lattice [the 4f and 5f electrons of the rare-earth (RE) elements and actinides, predominantly Ce, Yb, and U] such that the localized moments become effectively screened. More than 20 HF metals exhibit unconventional superconductivity, which does not obey the predictions of the Bardeen–Cooper–Schrieffer theory. HF metals are also prototype systems to explore the quantum critical point, with the most prominent examples being Ce- and Yb-containing compounds.^1^

In a search for unconventional HF compounds, we considered the case where the localized moments are distributed on a quasiperiodic lattice instead of a conventional distribution on a regular, translationally periodic lattice. The unconventional HF behavior in quasiperiodic structures is expected to occur from the “critical” character of the electronic wave functions, which are neither extended nor localized, but decay as a power law of the distance, ψ ∝ 1/*r*^*n*^. This is in contrast to the periodic crystals, where the wave functions are Bloch-type, extended states. It is an open question whether the critical wave functions still produce the Kondo effect that is at the origin of the HF phenomenon. One physical realization of the quasiperiodic distribution of localized moments is the RE-containing icosahedral quasicrystals (i-QCs) that possess the structure of the i-Cd_5.7_Yb parent compound. This structure is described as a quasiperiodic packing of interpenetrating rhombic triacontahedral atomic clusters (also named Tsai-type),^2−4^ where each rhombic triacontahedral cluster contains inside a three-shell icosahedral cluster, consisting of an inner dodecahedron, a middle icosahedron, and an outer icosidodecahedron. The middle icosahedron is populated by the RE atoms only, whereas there are no RE atoms on other shells. In the i-Cd_5.7_Yb-type structure, about 70% of the RE atoms are located on the icosahedra, whereas the remaining 30% are located in the “glue” atoms that fill the gaps between the clusters. The RE atoms in the glue are not distributed quasiperiodically, thus somewhat obscuring the physics of the quasiperiodically distributed localized moments.

Periodic approximants of the Tsai-type i-QCs with the general formula Cd_6_M are another type of structure with the localized moments distributed on icosahedra.^5^ The Cd_6_M structures (M = Pr, Nd, Sm, Eu, Gd, Dy, Yb, Y, and Ca) are based on periodic packing of the same rhombic triacontahedral cluster on a body-centered-cubic (bcc) lattice. In the approximants, all RE atoms are located exclusively on the icosahedra (i.e., there is only one RE crystallographic site), making a clear situation of quasiperiodically distributed localized spins. The prototype structures are Cd_6_Y, Cd_6_Yb, and Be_17_Ru_3_ (equivalent to Zn_17_Sc_3_). The skeletal networks of these three types of structures are identical, all consisting of the bcc packing of the interpenetrating rhombic triacontahedral clusters, whereas the difference comes from the species residing inside the central dodecahedral cavity. In Be_17_Ru_3_, the cavity is empty, whereas in the other two prototype structures, it contains a Cd_4_ tetrahedron exhibiting different types of disorder. In Cd_6_Yb, the disorder is modeled by a cube with half-occupancy of all vertices, whereas in Cd_6_Y, it is modeled by an icosahedron with one-third occupancy of all vertices.

Several RE-containing Tsai-type approximants have been reported so far in the literature, including 1/1 Cd_6_Yb,^6^ 2/1 Cd_5.8_Yb,^7^ 1/1 Zn_85.5_Sc_11_Tm_3.5_,^8^ 1/1 Ag_40_In_46_Yb_14_,^9^ 2/1 Ag_41_In_44_Yb_15_,^10^ 1/1 Gd_3_Au_13_Sn_4_,^11^ and 1/1 Ce_3_Au_13_Sn_4_.^12^ Apart from Ce_3_Au_13_Sn_4_, no other Ce-containing Tsai-type approximants were studied. The Ce element is exceptional because, being at the beginning of the RE series (having one 4f electron), the spatial extent of its 4f wave function is the largest, so that the exchange interaction between the 4f and conduction electrons is the strongest. Ce compounds are thus a natural choice to look for new HF Tsai-type approximants. In the previous research of the RE_3_Au_13_Sn_4_ system by some of our authors, the Ce_3_Au_13_Sn_4_ and isostructural La_3_Au_13_Sn_4_ compounds could not be grown as single-phase samples of large enough volume to be suitable for determination of their physical properties. Upon replacement of Sn by Ge, the results were better, and we performed Czochralski pulling of a Ce_3+*x*_Au_13+*y*_Ge_4+*z*_ crystal. We found that it is a stable Tsai-type 1/1 approximant with the composition Ce_3.2_Au_13.5_Ge_5.1_. In this paper, we report on its structural model and physical properties, with emphasis on possible HF behavior.

## 2 Structural Model

Experimental details of crystal growth by the Czochralski method are given in the Experimental Section. The structure was determined by single-crystal X-ray diffraction (XRD) using a Mo Kα X-ray source (the XRD experimental setup is also described in the Experimental Section). The structure was solved in the centrosymmetric space group *Im*3̅ (No. 204). All Au atomic positions, the Ce atomic position in 24g, and the Ge in 12e were obtained by direct methods, whereas the Ge(24g), Ge(8c), and Ce(2a) positions were obtained by difference Fourier synthesis. Before the last Ge atoms in 24g were added, the reliability factors were R1 = 2.9% and wR2 = 6.8%, with the largest electronic hole of 11.6 e Å^–3^. After this last position was added and the occupation factor was included in the refinement, the factors dropped to 2.3%, 4.6%, and 7.7 e Å^–3^, respectively.

The assignment of the crystallographic sites to the corresponding atoms has been performed by taking into account the isotropic displacement parameters for each position. For the positions 16f (Au4/Ge4) and 24g (Au5/Ge5), the analysis revealed mixed occupation by Ge and Au, with a total occupancy of 1. In the case of the positions 2a (Ce2) and 24g (Ge3), only one type of atom has been assigned to each position, with a partial occupancy in both cases. Actually, assignment of the Ce atoms to position 2a has been done, in agreement with the studies performed on Ca–Au–Ge and Yb–Au–Ge^13^ and Tb–Au–Si.^14^ The refinement with the 2a position occupied by Au atoms instead of Ce atoms has led to exactly the same reliability factor and the same residual values, with obviously smaller occupation by Au, but the result obtained with pure Ce was more in agreement with energy-dispersive spectroscopy (EDS) measurements. The same reason governed the decision to assign the 24g position to Ge3 only, without Au. Moreover, the smaller interatomic distances of the respective polyhedra (the icosahedra) do not appear to be compatible with large atoms. The complete crystallographic data are available in Table 1 (also provided as supplementary crystallographic data in CIF format), the final atomic coordinates and isotropic displacement parameters are given in Table 2, and the anisotropic displacement parameters are given in Table 3 of the Supporting Information.

**Table 1:** X-ray Crystallographic Data for Ce_3+*x*_Au_13+*y*_Ge_4+*z*_ (*x* = 0.17, *y* = 0.49, *z* = 1.08)

chemical formula	Ce_3.17(3)_Au_13.49(18)_Ge_5.08(30)_
fw (g mol^–1^)	3470.0
temperature (K)	296(2)
wavelength (Å)	0.71073
cryst size (μm^3^)	3 × 6 × 9
system	cubic
space group	*Im*3̅
unit cell dimension (Å)	*a* = 14.874(3)
volume (Å^3^)	3291.0(2)
*Z*/Pearson symbol	8/*cI*174
calcd density (g cm^–3^)	13.967
abs coeff (mm^–1^)	137.19
*F*(000)	11302
θ range for data collection (deg)	1.94–35.34
index ranges	–23 ≤ *h* ≤ 23, −23 ≤ *k* ≤ 23, −23 ≤ *l* ≤ 24
collected reflns, indep reflns, >2σ	49127, 1328, 1159
coverage of the reciprocal sphere (%)	97.3
GOF	1.039
*R* indices	*R*_int_ = 0.08, R1 = 0.023, wR2 = 0.046
extinction coeff	0.0000302(17)
no. of refined param	53
Δρ_max_/Δρ_min_ (e Å^–3^)	7.701/–3.774

**Table 2:** Atomic Coordinates and Isotropic Displacement Parameters for Ce_3.17_Au_13.49_Ge_5.08_

atom name	site	*x*	*y*	*z*	*U*_eq_ (Å^2^)	occupancy
Ce1	24g	0.30215(3)	0.18621(3)	0	0.00683(9)	1
Ce2	2a	0	0	0	0.0313(12)	0.696(13)
Au1	24g	0.35482(2)	0.40290(2)	0	0.00944(7)	1
Au2	48h	0.20006(2)	0.34121(2)	0.10543(2)	0.01256(7)	1
Au3	12d	^1^/_2_	^1^/_2_	0.90582(4)	0.01580(11)	1
Au4/Ge4	16f	0.14416(4)	*x*	*x*	0.0315(3)	0.769(5)/0.231(5)
Au5/Ge5	24g	0.08449(4)	0.23440(4)	0	0.01236(17)	0.486(4)/0.514(4)
Ge1	12e	^1^/_2_	0.30122(10)	0	0.0124(3)	1
Ge2	8c	^1^/_4_	^1^/_4_	^1^/_4_	0.0388(7)	1
Ge3	24g	0.0634(5)	0.0915(5)	0	0.029(2)	0.192(6)

The refined composition is Ce_25.39(0.24)_Au_107.68(1.44)_Ge_40.88(2.4)_, corresponding to Ce_3.17(0.03)_Au_13.49(0.18)_Ge_5.08(0.30)_ or, in atomic percent, to Ce_14.6_Au_61.9_Ge_23.5_, which is close to the starting composition (see the Experimental Section). In the following, we shall refer to this material by its generic (stoichiometric) chemical formula Ce_3_Au_13_Ge_4_. EDS measurements performed on different samples revealed a broad homogeneity range of the Ce_3_Au_13_Ge_4_ phase, extending from Ce_3_Au_13_Ge_2.7_ to Ce_3.25_Au_14.2_Ge_5.1_. Similar to the Ce–Au–Sn system,^15^ this phase region is in equilibrium with the 1–2–2 ternary phase (i.e., CeAu_2_Ge_2_, structure type CeAl_2_Ga_2_, with space group *I*4/*mmm*), which has also been detected in the powder XRD pattern of our investigated sample (not shown here), with the estimated fraction of less than 4% (in atomic percent).

**Figure 1:**
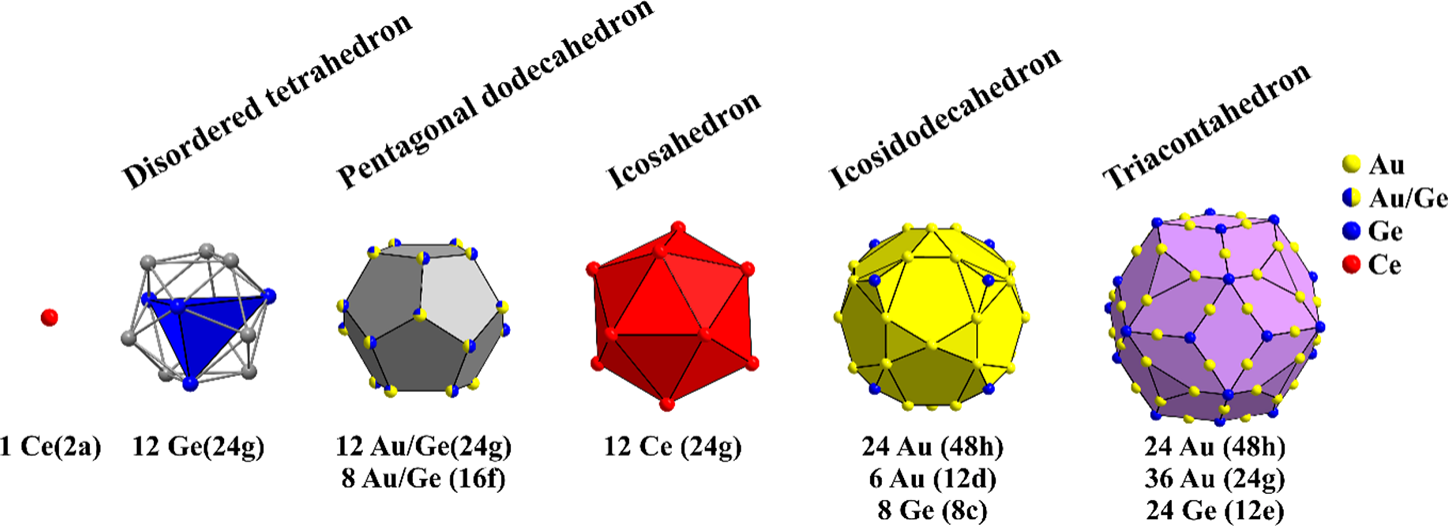
Successive atomic shells of the multishell atomic cluster as the basic building block of the Ce_3_Au_13_Ge_4_ structure.

The basic building block of the structure is a multishell atomic cluster, depicted in Figure 1.

From inside out, the cluster center contains one partially populated Ce site (occupation 0.7), which resides in the center of a statistically disordered Ge_4_ tetrahedron, modeled by an icosahedron with 0.192 occupancy of all vertices (corresponding to 2.4 atoms per icosahedron). The Ge_4_ tetrahedron resides inside an (Au,Ge)_20_ pentagonal dodecahedron with mixed-occupied Au/Ge sites, which is surrounded by a Ce_12_ icosahedron. The next shell is a 38-atom polyhedron, consisting of an Au_30_ icosidodecahedron with eight additional Ge atoms in a cubic arrangement, located above the centers of the triangular faces, as shown in Figure 1. The outermost shell is an (Au,Ge)_84_ triacontahedron.

**Figure 2:**
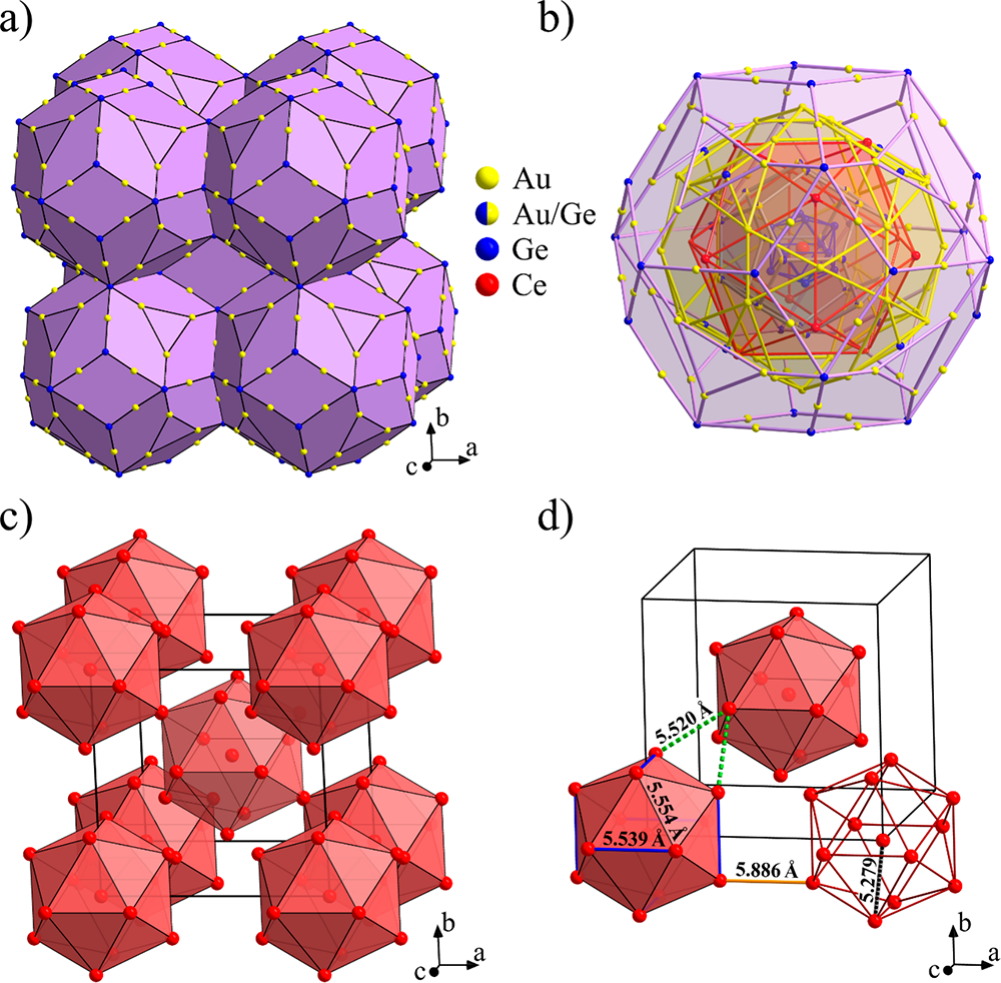
(a) Unit cell of the Ce_3_Au_13_Ge_4_ structure. (b) One complete triacontahedral cluster, showing the successive atomic shells. (c) Ce magnetic sublattice. (d) Interspin distances on the Ce sublattice.

The structure is described as a bcc packing of partially interpenetrating triacontahedral clusters, as shown in Figure 2a, whereas one complete triacontahedral cluster is shown in Figure 2b. The lattice parameter of the bcc unit cell is *a* = 14.874 Å, and the cell contains 194 lattice sites, out of which 173.9 are occupied by atoms (Pearson symbol *cI*174) because of partial occupation of the Ce2 and Ge3 sites. There are no “glue” atoms between the triacontahedral clusters. The Ce sublattice, corresponding to the magnetic sublattice, is shown in Figure 2c. It consists of a bcc packing of Ce icosahedra with an additional atom in a (partially occupied) Ce2 site at the center of the icosahedron.

The above-described structure of the Ce_3+*x*_Au_13+*y*_Ge_4+*z*_ is very similar to the other Cd_6_M Tsai-type approximants. The main difference is the additional RE atom in the cluster center (Ce2 site of a 0.7 partial occupation) and the eight additional (Ge) atoms in a cubic arrangement on the Au_30_ icosidodecahedral shell. Similar structural arrangements have been reported also for related ternary systems with distinct disorder between the Au and semimetallic elements like (Yb,Gd)–Au–(Si,Ge),^16^ (Ca,Yb)–Au–Sn,^17^ and Yb–Au–Ga.^18^

## 3 Physical Properties

### 3.1 Magnetization and Magnetic Susceptibility

**Figure 3:**
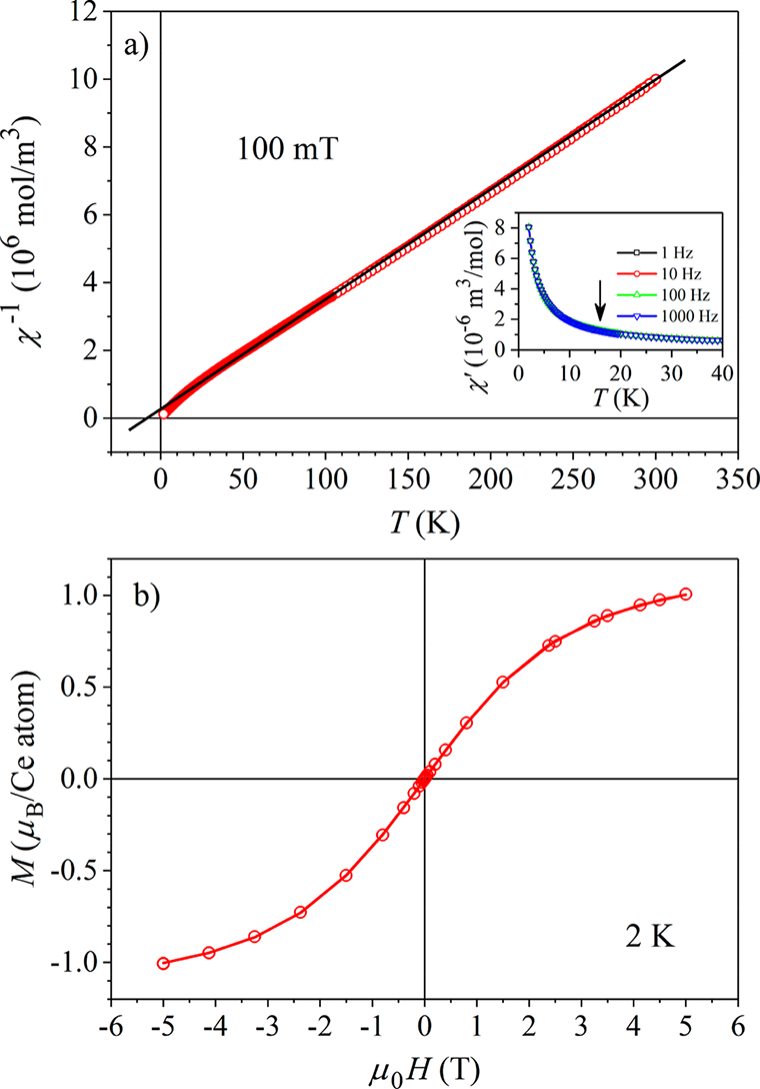
(a) Inverse magnetic susceptibility χ^–1^ in a magnetic field μ_0_*H* = 100 mT. The solid line is the Curie–Weiss fit for temperatures *T* > 50 K. The inset shows the real part of the ac susceptibility χ′ between 40 and 2 K at frequencies of 1, 10, 100, and 1000 Hz. The vertical arrow at 16 K indicates the temperature of the AFM transition in bulk CeAu_2_Ge_2_ (see the text). (b) Magnetization versus the magnetic field, *M*(*H*), at *T* = 2 K. The solid curve is the fit with eq 2.

The direct-current (dc) magnetic susceptibility χ = *M*/*H* was determined in the temperature range 1.9–300 K in magnetic field μ_0_*H* = 100 mT. The inverse susceptibility χ^–1^, corrected for the diamagnetic contribution due to core electrons, is shown in Figure 3a. The high-temperature data (for *T* > 50 K) were analyzed with the Curie–Weiss law
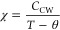
1and the fit is shown by a solid line in Figure 3a. The fit yielded the Curie–Weiss constant *C*_CW_ = 3.15 × 10^–5^ K·m^3^/mol, and the Curie–Weiss temperature θ = −7 K. The effective magnetic moment per Ce atom calculated from *C*_CW_ was determined to be μ_eff_ = 2.58 μ_B_, where μ_B_ is the Bohr magneton. This μ_eff_ value is practically identical with the theoretical Ce^3+^ free-ion value μ_Ce_ = 2.54 μ_B_. The inverse susceptibility shows a slight deviation from the Curie–Weiss fit below 10 K, but there is no indication of any kind of phase transition down to the lowest measured temperature of 1.9 K. The susceptibility analysis indicates that the material is paramagnetic down to 1.9 K, the Ce moments are localized, and the moments’ value for *T* > 50 K equals the Ce^3+^ free-ion value, whereas the negative θ indicates indicates antiferromagnetic (AFM) correlations between the Ce spins.

Because XRD has demonstrated the presence of a small amount of CeAu_2_Ge_2_ inclusions in the Ce_3_Au_13_Ge_4_ matrix, which (in a bulk volume) undergo a transition to the AFM state at the Néel temperature *T*_N_ = 16 K,^19^ we inspected the dc susceptibility around that temperature, but no trace of an anomaly could be found. To check further for the possible AFM magnetization component with the phase transition at 16 K, we measured the alternating-current (ac) susceptibility at frequencies of 1, 10, 100, and 1000 Hz. The real part of the ac susceptibility χ′ between 40 and 1.9 K is shown in the inset of Figure 3a. No trace of an AFM transition at 16 K could be noticed in χ′, indicating that the CeAu_2_Ge_2_ inclusions do not yield any observable AFM magnetization component (perhaps the inclusions’ volumes are too small to undergo the AFM transition).

The magnetization versus magnetic field relationship, *M*(*H*), was determined at *T* = 2 K and is shown in Figure 3b. The data were analyzed by the function
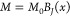
2where *M*_0_ is the saturation magnetization, *B*_*J*_(*x*) with *x* = μ_0_*gJ*μ_B_*H*/*k*_B_*T* is the Brillouin function, describing the response of localized paramagnetic moments of the angular momentum ℏ to the external magnetic field μ_0_*H*, and *g* is the Landé factor. Equation 2 is derived from the Zeeman Hamiltonian . In the fit procedure, *M*_0_ and *g* were taken as free parameters, whereas *J* was fixed to ^5^/_2_. An excellent fit (solid curve in Figure 3b) was obtained with the fit parameters *M*_0_ = 1.13 μ_B_/Ce atom and *g* = 0.87. This *g* value is practically identical with the theoretical Ce^3+^ free-ion value of 0.86. The fit-determined saturation magnetization *M*_0_ is, however, considerably smaller than the theoretical value for noninteracting Ce spins, which amounts to *M*_0_ = *gJ*μ_B_ = 2.15 μ_B_/Ce atom. The reduction of *M*_0_ is a consequence of the AFM interspin interactions that are already significant at *T* = 2 K (as evidenced by the Curie–Weiss deviation of χ^–1^ from the straight line in Figure 3a below 10 K). The *M*(*H*) curve does not show any hysteresis at 2 K, supporting that the sample is still in the paramagnetic phase at that temperature, but short-range AFM correlations between the spins are already present.

### 3.2 Specific Heat

**Figure 4:**
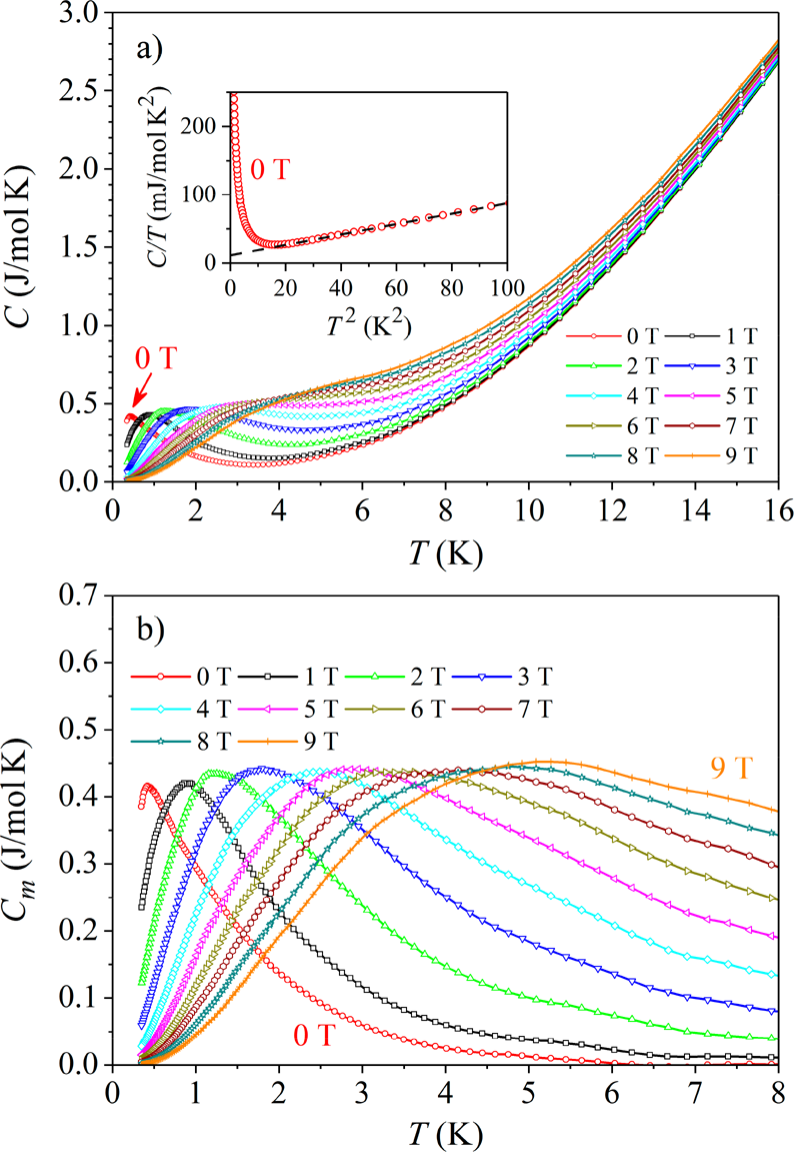
(a) Low-temperature total specific heat between 0.35 and 16 K in magnetic fields between 0 and 9 T. The inset shows the zero-field specific heat between 0.35 and 10 K in a *C*/*T* versus *T*^2^ plot. The dashed line is the fit with the expression *C*/*T* = γ + α*T*^2^. (b) Magnetic specific heat *C*_m_ = *C* – γ*T* – α*T*^3^ versus *T* in the temperature interval between 0.35 and 8 K in magnetic fields up to 9 T.

Specific heat *C* was measured between 0.35 and 300 K in magnetic fields 0–9 T in steps of 1 T. The low-temperature specific heat between 0.35 and 16 K is shown in Figure 4a. A maximum is observed at the lowest investigated temperatures, which broadens and shifts to higher temperatures with the increasing field. The zero-field specific heat at temperatures between 0.35 and 10 K in a *C*/*T* versus *T*^2^ plot is shown in the inset of Figure 4a, where it is seen that away from the maximum, the data fall on a straight line.

The low-temperature specific heat was analyzed by the expression
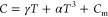
3where γ*T*and α*T*^3^ are the electronic and lattice contributions, respectively, whereas *C*_m_ is the magnetic specific heat. The fit of the zero-field data away from the maximum with the expression *C*/*T* = γ + α*T*^2^ (dashed line in the inset of Figure 4a) yielded the electronic specific heat coefficient γ = 11.3 mJ/mol·K^2^ and the Debye temperature (extracted from the lattice specific heat coefficient α) θ_D_ = 137 K. The corresponding γ values for the Ce and Au metals are γ_Ce_ = 12.8 mJ/mol·K^2^ and γ_Au_ = 0.69 mJ/mol·K^2^ (Ge is a semiconductor), so that γ of Ce_3_Au_13_Ge_4_ is very close to that of the Ce metal. The γ coefficient does not show an enhancement characteristic of the HF systems, where γ values on the order of 1 J/mol·K^2^ are common, revealing that the conduction electrons are not “heavy” and Ce_3_Au_13_Ge_4_ is a regular intermetallic compound with no resemblance to HF systems.

The electronic and lattice contributions were subtracted from the total specific heat to obtain the magnetic specific heat *C*_m_ = *C* – γ*T* – α*T*^3^. A plot of *C*_m_ versus *T* in the temperature interval between 0.35 and 8 K in magnetic fields up to 9 T is shown in Figure 4b. The zero-field *C*_m_ shows a well-pronounced maximum at *T*_max_ ≈ 0.4 K and decays at higher temperatures, becoming zero at about 6 K. In an increasing magnetic field, the maximum broadens and shifts to higher temperatures, whereas the decay slows down, so that the magnetic specific heat remains nonzero to higher temperatures.

The observed temperature and field dependence of *C*_m_ is typical of a spin glass and/or other magnetically frustrated spin systems that undergo a spin freezing transition at the temperature *T*_f_. The magnetic specific heat of these systems passes through a broad peak at a temperature greater than the spin freezing temperature *T*_f_ (typically at *T*_max_ ∼ 1.4*T*_f_^20^) and then starts to decrease slowly upon heating. The external field broadens the peak and shifts it to higher temperatures. The temperature-dependent decrease in *C*_m_ above the maximum also slows down as the temperature is raised further, so that *C*_m_ in a higher field is larger. This is exactly what is observed in Figure 4b. Because the temperature of the peak amounts to *T*_max_ ≈ 0.4 K, this gives an estimate of the spin freezing temperature of *T*_f_ ≈ 0.28 K. In our experiments conducted down to 0.35 K, we are thus observing the spin-glass-type specific heat *C*_m_ in its high-temperature regime above the spin freezing temperature *T*_f_, without actually entering the broken-ergodicity collective magnetic state of a frustrated spin system.

## 4 Discussion and Conclusions

In a search for unconventional HF compounds with the localized 4f moments distributed quasiperiodically, we have successfully synthesized a stable Ce_3_Au_13_Ge_4_ Tsai-type 1/1 quasicrystalline approximant and determined its structural model. Despite the expectation that the compound might exhibit HF properties, the measurements of its magnetic properties and the specific heat have demonstrated that it is a regular intermetallic compound with no resemblance to HF systems. Magnetically, Ce_3_Au_13_Ge_4_ is quite similar to the formerly investigated Gd_3_Au_13_Sn_4_,^11^ where a spin-glass phase with the spin freezing temperature *T*_f_ ≈ 2.8 K was observed and the spin-glass behavior was attributed to the geometric frustration of the AFM-coupled Gd moments placed on equilateral triangles of the icosahedral magnetic sublattice. In Ce_3_Au_13_Ge_4_, the Ce moments are also AFM-coupled, as evidenced from the negative value of the Curie–Weiss temperature. Geometric frustration of the spin system is present as well, as seen from the distance analysis of the Ce sublattice, presented in Figure 2d. The 12 Ce1 atoms of the icosahedron are distributed on triangles with side lengths of 5.539 Å (6 sides) and 5.554 Å (24 sides), so that the triangles are almost equilateral. Another triangular distribution of spins on triangles of very similar dimensions is formed by the Ce atoms of the central icosahedron and the nearest-neighbor Ce atoms of the icosahedra located at the vertices of the bcc unit cell (dashed bonds in Figure 2d). These triangles are again almost equilateral, with two sides of 5.520 Å length and one side of 5.554 Å. In addition to the geometric frustration, the Ce magnetic lattice contains also randomness due to the partially occupied Ce2 site (occupation 0.7) in the center of each Ce1 icosahedron (this central spin is absent in the Gd magnetic lattice of the Gd_3_Au_13_Sn_4_ structure). The Ce2–Ce1 distance of 5.279 Å is the shortest Ce–Ce distance in the structure, so that randomly distributed Ce2 vacancies are expected to have a strong impact on the collective magnetic state. Other sources of randomness are the mixed-occupied Au/Ge sites (the sites Au4/Ge4 and Au5/Ge5) of the pentagonal dodecahedral shell, which are the ligand sites between the Ce2 central atom and Ce1 icosahedron, and the random compositional fluctuations due to nonstoichiometry of the investigated Ce_3+*x*_Au_13+*y*_Ge_4+*z*_ alloy, which introduce substitutional (chemical) disorder. Randomness causes a distribution of the RKKY indirect-exchange interactions between the spins and is considered to be the dominant origin of the spin-glass phenomenon in the Ce_3_Au_13_Ge_4_ approximant. Geometric frustration due to the triangular distribution of the Ce moments adds to the spin-glass behavior as well.

The difference in the spin freezing temperatures of the Gd_3_Au_13_Sn_4_ and Ce_3_Au_13_Ge_4_ quasicrystalline approximants can be related to the magnitude of the magnetic moments, where the Gd paramagnetic moment μ_Gd_ = 7.94 μ_B_ is by a factor of 3.1 larger than the Ce moment μ_Ce_ = 2.54 μ_B_. The larger Gd moments in a spin-glass configuration are interacting stronger and undergo a spin freezing transition at *T*_f_ ≈ 2.8 K, whereas for the considerably smaller Ce moments, the interactions are weaker and the spin freezing transition is shifted to a lower temperature of *T*_f_ ≈ 0.28 K.

## 5 Experimental Section

The source material was an alloy Ce_15_Au_65_Ge_20_ (in atom %), produced by arc melting under an Ar atmosphere. The obtained button was annealed at 700 °C for 14 days. From this alloy, a crystal was grown by the Czochralski technique using a Cyberstar apparatus. The detailed description of the instrument and the procedure used for single-crystal growth can be found in our recent paper.^21^

The single-crystal XRD data were collected on a Bruker Kappa Apex II diffractometer equipped with a mirror monochromator and a Mo Kα IμS (λ = 0.71073 Å). The *Apex2* program package was used for cell refinement and data reduction. The structure was solved by using direct methods and refined with the *SHELXL-2013* program. Semiempirical absorption correction (*SADABS*) was applied to the data.

Magnetic measurements were conducted on a Quantum Design MPMS XL-5 SQUID magnetometer equipped with a 5 T magnet, operating in the temperature range 1.9–400 K. The specific heat was measured on a Quantum Design Physical Property Measurement System (PPMS 9T), equipped with a 9 T magnet and a ^3^He cryostat, operating in the temperature range 0.35–400 K.

## Supporting Information Available

Anisotropic atomic displacement parameters (Å^2^) of the Ce_3+*x*_Au_13+*y*_Ge_4+*z*_ (*x* = 0.17, *y* = 0.49, *z* = 1.08) structural model (PDF)

## Accession Codes

CCDC 2045347 contains the supplementary crystallographic data for this paper. These data can be obtained free of charge via www.ccdc.cam.ac.uk/data_request/cif, or by emailing data_request@ccdc.cam.ac.uk, or by contacting The Cambridge Crystallographic Data Centre, 12 Union Road, Cambridge CB2 1EZ, UK; fax: +44 1223 336033.
