# Supplementary material for: Structural Model and Spin-Glass Magnetism of the Ce3Au13Ge4 Quasicrystalline Approximant
Source: Inorg Chem. 2021 Feb 3;60(4):2526–32. doi: 10.1021/acs.inorgchem.0c03430 (PMC8827497; doi:10.1021/acs.inorgchem.0c03430)
Supplement: Supplementary file 1 — ic0c03430_si_001.pdf [file ic0c03430_si_001.pdf]

Supporting Information

Structural model and spin-glass magnetism of the $Ce_3Au_{13}Ge_4$ quasicrystalline approximant

Pascal Boulet,[1,§] Marie-Cécile de Weerd,[1] Mitja Krnel,[2] Stanislav Vrtnik,[2] Zvonko Jagličić,[3,4] Janez Dolinšek[2,5,*]

[1] *Institut Jean Lamour, UMR 7198 CNRS – Université de Lorraine, Campus Artem, 2 allée André Guinier, BP 50840, 54011 Nancy Cedex, France*

[2] *J. Stefan Institute, Jamova 39, SI-1000 Ljubljana, Slovenia*

[3] *Institute of Mathematics, Physics and Mechanics, Jadranska 19, SI-1000 Ljubljana, Slovenia*

[4] *University of Ljubljana, Faculty of Civil and Geodetic Engineering, Jamova 2, SI-1000 Ljubljana, Slovenia*

[5] *University of Ljubljana, Faculty of Mathematics and Physics, Jadranska 19, SI-1000 Ljubljana, Slovenia*

* Corresponding author. *E-mail address*: jani.dolinsek@ijs.si (J. Dolinšek).

§ Corresponding author. *E-mail address*: p.boulet@univ-lorraine.fr (P. Boulet).

Table 3. Anisotropic atomic displacement parameters (Å^2) of the $Ce_{3.17}Au_{13.49}Ge_{5.08}$ structural model.

	U11	U22	U33	U23	U13	U12
Ce1	0.00797(19)	0.00487(18)	0.00764(19)	0	0	0.00081(15)
Ce2	0.0313(12)	0.0313(12)	0.0313(12)	0	0	0
Au1	0.01106(15)	0.00851(14)	0.00877(14)	0	0	-0.00017(11)
Au2	0.01080(11)	0.01117(11)	0.01570(12)	-0.00344(8)	0.00316(8)	-0.00037(8)
Au3	0.0078(2)	0.0315(3)	0.0081(2)	0	0	0
Au4/Ge4	0.0315(3)	0.0315(3)	0.0315(3)	0.0157(2)	0.0157(2)	0.0157(2)
Au5/Ge5	0.0121(3)	0.0170(3)	0.0080(2)	0	0	0.00442(19)
Ge1	0.0065(6)	0.0115(6)	0.0193(7)	0	0	0
Ge2	0.0388(7)	0.0388(7)	0.0388(7)	0.0165(9)	0.0165(9)	0.0165(9)
Ge3	0.035(4)	0.023(4)	0.027(4)	0	0	-0.019(3)
